# The prognostic value of vascular endothelial growth factor in endometrial cancer: A protocol for systematic review and meta-analysis

**DOI:** 10.1097/MD.0000000000040933

**Published:** 2024-12-20

**Authors:** Bao Qiang, YiFan Kang, JiaoLin Yang, HuanCheng Su, Zhe Wang, ChunMei Zhang, SanYuan Zhang

**Affiliations:** a Shanxi Medical University, Taiyuan, Shanxi Province, China; b First Hospital of Shanxi Medical University, Taiyuan, Shanxi Province, China.

**Keywords:** endometrial cancer, endothelial growth factor, meta-analysis, prognosis, survive, vascular

## Abstract

**Background:**

A large number of studies have shown that high expression of vascular endothelial growth factor (VEGF) in cancer tissues is associated with poor prognosis of various cancers. However, this finding in endometrial cancer is controversial. Therefore, this meta-analysis aimed to explore the effects of VEGF on survival in patients with endometrial cancer.

**Methods:**

Four databases of PubMed, Medline, Web of Science, and China National Knowledge Infrastructure were searched to collect literature that met the inclusion criteria. The association between high VEGF expression and survival outcomes and clinicopathological features of patients with cancer was evaluated by calculating the combined hazard ratio (HR), odds ratio (OR), and 95% confidence interval (CI). The Begg test was used to assess publication bias.

**Results:**

A total of 11 studies were included, involving 1251 patients. The results showed that compared with low VEGF expression, high VEGF expression was significantly associated with shorter overall survival (HR = 2.44, 95% CI = 1.15–5.16, I^2^ = 80%, *P* = .02) and disease-specific survival (HR = 7.87, 95% CI = 1.70–36.44, I^2^ = 64%, *P* = .008) but not with disease-free survival (HR = 1.45, 95% CI = 0.70–3.02, I^2^ = 68%, *P* = .32). In addition, VEGF expression is higher in patients with advanced stage (OR = 3.70, 95% CI = 2.22–6.19, *P *< .001), lower histological differentiation (OR = 2.08, 95% CI = 1.22–3.55, *P* = .007), and lymph node metastasis (OR = 5.42, 95% CI = 2.35–5.11, *P *< .001).

**Conclusion:**

High VEGF expression can predict poor prognosis and poor clinicopathological features in patients with endometrial cancer, and it may be a valuable new indicator to evaluate the prognosis of patients with endometrial cancer.

## 1. Introduction

Endometrial cancer is one of the most common malignant tumors in women.^[1]^ The number of patients with endometrial cancer in the United States has been reported to be increasing from 2015 to 2019, with an estimated 66,200 new cancer cases and 13,030 deaths in 2023.^[2]^ In recent years, although the 5-year survival rate of endometrial cancer has been well improved, the risk of recurrence and death is still very high, especially for advanced patients, and even in early patients, the recurrence rate can reach 30%,^[3]^ resulting in poor prognosis of patients. With the development of molecular biology, molecular typing plays a significant guiding role in the treatment and prognosis of endometrial cancer.^[4]^ However, due to the expensive and time-consuming nature of these techniques, their application is limited. Therefore, there is a need to find new simpler and more accurate biomarkers to predict the prognosis of endometrial cancer and to develop new therapeutic targets to improve patient outcomes.

Vascular endothelial growth factor (VEGF) is a highly conserved homologous dimer glycoprotein, whose gene is localized to human chromosome 6p21.3, and it is a specific factor promoting angiogenesis.^[5]^ It is well known that angiogenesis is a key factor in the invasion and metastasis of malignant tumors, and VEGF plays a central role in this process.^[6]^ Studies have shown that VEGF levels are significantly increased in a variety of malignant tumors (such as prostate cancer,^[7]^ gastric cancer,^[8]^ colorectal cancer,^[9]^ etc) and are closely related to invasion and metastasis and poor prognosis. In recent years, many studies have analyzed the prognostic value of VEGF in endometrial cancer. Zinovkin et al^[10]^ have shown that VEGF plays a negative role in the survival of patients with endometrial cancer, and some researchers have shown that VEGF is not associated with poor prognosis of patients with endometrial cancer.^[11,12]^ Thus, the role of VEGF in the prognosis of endometrial cancer is still controversial.

Therefore, we used systematic review and meta-analysis methods to explore the correlation between VEGF and endometrial cancer survival, aiming to determine the prognostic value of high VEGF expression in endometrial cancer and help guide clinical follow-up and treatment.

## 2. Materials and methods

This systematic review and meta-analysis was conducted in accordance with the Preferred Reporting Project Statement for Systematic Reviews and Meta-Analyses. It has been registered in the International Prospective Register of Systematic Reviews (CRD42024504321).

### 2.1. Search strategy

We searched 4 databases, PubMed, Medline, Web of Science, and China National Knowledge Infrastructure, to find articles involving VEGF and endometrial cancer prognosis published in each database from inception to December 2023. The search terms used are as follows: “vascular endothelial growth factor or VEGF and endometrial cancer or “endometrium cancer” or “endometrium carcinoma” or “endometrial neoplasms” or “endometrial carcinoma” or “cancer of endometrium” or “cancer of the endometrium” or “carcinoma of endometrium”. We have no restrictions on language, but animal research is excluded. Differences encountered during the search are determined after discussion with other authors.

### 2.2. Inclusion and exclusion criteria

Inclusion criteria were as follows: endometrial cancer was confirmed by histopathology; the study measured the expression level of VEGF in tissues after endometrial cancer surgery (positive/high, negative/low); hazard ratio (HR) and 95% confidence interval (CI) data for overall survival (OS), disease-free survival (DFS), and disease-specific survival (DSS) were reported or there was a survival curve in the study; the study included the relationship between VEGF and clinicopathological features. Exclusion criteria were as follows: reviews, meta-analyses, case reports, or abstracts of meetings; animal researches or cell experiments; studies where data cannot be extracted; replicated study.

### 2.3. Quality evaluation

The quality of each included article was assessed using the Newcastle-Ottawa Scale (NOS), which consisted of 3 components: selection, comparability, and outcome (with a total score of 4, 2, and 3, respectively). NOS scores ≥6 were classified as high-quality studies and NOS scores <6 as low-quality studies.

### 2.4. Data extraction

Data were extracted independently by 2 authors (Bao Qiang and YiFan Kang), and the information collected included author, year, country, sample source, sample size, detection method, treatment method, clinicopathological features (tumor node metastasis stage, histological grade, depth of myometrium invasion, lymph node metastasis), follow-up duration, HR, and 95% CI. If both univariate and multivariate results are reported in the study, we choose the latter. If there is a survival curve in the article but HR and 95% CI values are not reported directly, we use EngaugeDigitizer to extract data from the survival curve and calculate the HR and 95% CI values.

### 2.5. Statistical analysis

Meta-analysis was performed using the Review Manager 5.3 and Stata 15.0 softwares. Combined HR and 95% CI were used to evaluate the effect of VEGF expression on the prognosis of endometrial cancer. When HR (high/low) is >1, it indicates that patients with high VEGF expression have a worse prognosis. Odds ratio and 95% CI were used to assess the relationship between VEGF expression and clinicopathological outcomes. Statistical heterogeneity was assessed using the I^2^ test, when I^2^ >50%, there was heterogeneity, using the random-effects model, and when  I^2^ ≤50%, using the fixed-effects model. In the case of significant heterogeneity, further sensitivity analysis or subgroup analysis was used to find the source of heterogeneity. The sensitivity analysis is carried out by one-by-one elimination method. We used the Begg test to assess publication bias. *P* < .05 was considered statistically significant.

## 3. Results

### 3.1. Search results

**Figure 1.**
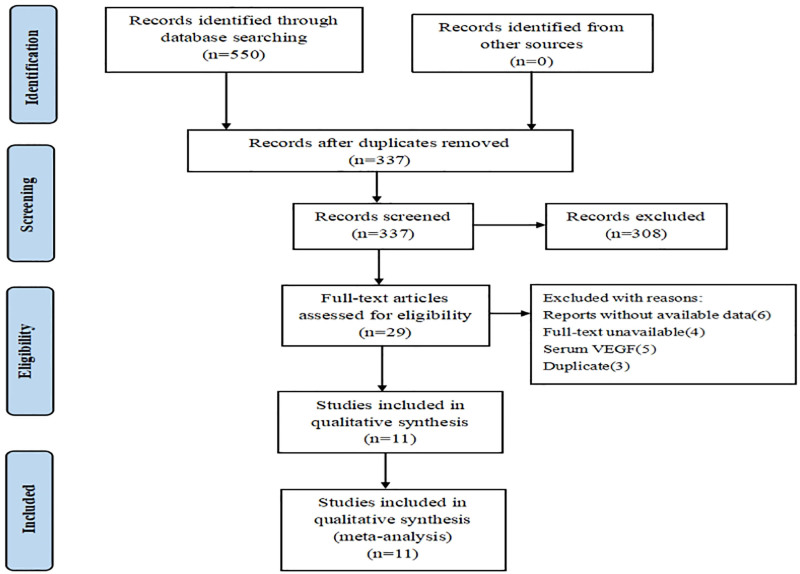
The flowchart of study selection. VEGF = vascular endothelial growth factor.

A total of 550 articles were initially obtained from the 4 databases, with 29 remaining articles after eliminating duplicates and reading titles and abstracts. By reading the full text, the final 11 articles were included in the study,^[10–20]^ involving a total of 1251 patients (Fig. 1). The reasons for the exclusion of 18 articles were as follows: 3 articles had duplicate data, 5 articles were about serum VEGF, 4 articles could not obtain the full text, and 6 articles could not obtain data because HR value and survival curve were not reported.

### 3.2. Features of the included study

Detailed characteristics of all eligible studies are shown in Table 1. The 11 studies were published between 1999 and 2020, with 10 in English and 1 in Chinese. HR and 95% CI in 4 studies were derived from direct extraction, and HR and 95% CI in 7 studies were derived from survival curve calculations. Immunohistochemical methods were used to detect VEGF expression levels in all studies. Studies have shown that VEGF is related to patients’ OS, DFS, and DSS in 5, 2, and 3 articles, respectively, and there is also 1 article related to both OS and DFS. All included studies were of high quality (≥6 points) after the NOS score, as shown in Table 2.

**Table 1:** Features of included studies

Study	Year	Country	Sample size	Source of samples	Detection method	Means of treatment	Histological grade (G1-G2/G3)	Tumor stage (I-II/III-IV)	Myometrial invasion (≤½/>½)	Lymph node metastasis (no/yes)	Follow-up time (mo)	Outcomes
Fujiwaki et al	1999	Japan	50	Tissue	IHC	1 or 1 + 2/3/4	47/3	43/7	30/20	NP	4–168	DFS
Yokoyama et al	2000	Japan	86	Tissue	IHC	1	NP	55/31	NP	NP	NP	OS
Hirai et al	2001	Japan	228	Tissue	IHC	1	NP	176/52	NP	212/16	NP	DFS
Giatromanolaki et al	2001	The United States	121	Tissue	IHC	1 or 1 + 2	NP	107/14	57/64	121/0	4–182	OS
Yokoyama et al	2003	Japan	71	Tissue	IHC	1	61/10	39/32	48/23	45/26	8–198	DSS
Talvensaari-Mattila et al	2005	Finland	115	Tissue	IHC	1 or 1 + 2/3	102/13	98/17	82/33	NP	0–124	OS
Kamat et al	2007	The United States	111	Tissue	IHC	1	88/23	77/34	NP	NP	NP	DSS
Merritt et al	2010	The United States	85	Tissue	IHC	1 or 1 + 2/3	72/13	59/26	62/23	NP	NP	DSS
Topolovec et al	2010	Croatia	87	Tissue	IHC	1 or 1 + 2/3/4	71/16	NP	NP	NP	60–132	OS
Li et al	2018	China	197	Tissue	IHC	1	144/53	170/27	51/146	172/25	6–67	OS
Zinovkin et al	2019	UK	100	Tissue	IHC	1	87/13	71/29	NP	NP	36	OS, DFS

Means of treatment: 1, surgery; 2, radiation; 3, chemotherapy; 4, hormone therapy.

DFS = disease-free survival, DSS = disease-specific survival, IHC = immunohistochemistry, NP = not reported, OS = overall survival.

**Table 2:** Quality evaluation of the eligible studies with the Newcastle-Ottawa Scale

Study	Selection				Comparability		Outcome			
	Representativeness of exposed cohort	Selection of nonexposed cohort	Ascertainment of exposure	Outcome not present at start	Comparability of cohorts on the basis of the design or analysis	Assessment of outcome		Adequate follow-up length	Adequacy of follow-up	Overall
Fujiwaki et al	*	*	*	*	*		*	*	*	8
Yokoyama et al	*	*	*	*	*		*			6
Hirai et al	*	*	*	*			*		*	8
Giatromanolaki et al	*	*	*	*	*		*	*		7
Yokoyama et al	*	*	*	*	*		*	*	*	8
Talvensaari-Mattila et al	*	*	*	*			*	*	*	9
Kamat et al	*	*	*	*	*		*		*	7
Merritt et al	*	*	*	*	*		*		*	7
Topolovec et al	*	*	*	*	*		*	*		7
Li et al	*	*	*	*	*		*		*	7
Zinovkin et al	*	*	*	*	*		*	*	*	8

* 1 point.

### 3.3. Association between VEGF expression and prognosis of patients with endometrial cancer

#### 3.3.1. Overall survival

**Figure 2.**
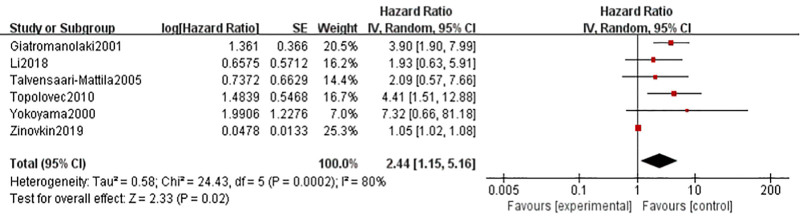
Forest map of the association between VEGF expression and OS of endometrial cancer. CI = confidence interval, IV = random effects model, OS = overall survival, SE = standard deviation, VEGF = vascular endothelial growth factor.

A total of 6 studies reported the association between high VEGF expression and OS in patients with endometrial cancer. As shown in Figure 2, due to high heterogeneity (I^2^ = 80%), we found that high VEGF expression was associated with shorter OS in patients with endometrial carcinoma using a random-effects model (HR = 2.44, 95% CI = 1.15–5.16, I ^2^ = 80%, *P* = .02). We further conducted subgroup analysis (Table 3). Grouping by year of publication, the results found that high levels of VEGF were significantly associated with worse outcomes in the 2000 to 2010 study (HR = 3.74, 95% CI, 2.20–6.34, I^2^ = 0.0%, *P *< .001); after the sample size was divided into 2 groups, in the large sample size group (>100), high expression of VEGF was a marker of poor prognosis (HR = 2.95, 95% CI = 1.71–5.10, I^2^ = 0.0%, *P* < .001); in a treatment-based subgroup analysis, high levels of VEGF expression were associated with poorer OS in combination therapy (HR = 3.61, 95% CI = 2.10–6.21, I^2^ = 0.0%, *P* < .001); in subgroup analysis by country, no correlation between high VEGF expression and prognosis was observed in either group (*P* > .05).

**Table 3:** Subgroup analysis of the correlation between high expression of VEGF and OS

Subgroups	No. of studies	No. of patients	Pooled HR (95% CI)	*P* value	Hewterogeneity		Model
					I^2^ (%)	*P* value	
Publication year							
2000–2010	4		3.74 (2.20–6.34)	<0.001	0	0.76	Fixed-effects model
>2010	2		1.09 (0.82–1.45)	0.56	12	0.29	Fixed-effects model
Sample size							
>100	3		2.95 (1.71–5.10)	<0.001	0	0.50	Fixed-effects model
≤100	3		2.46 (0.68–8.94)	0.17	79	0.009	Random-effects model
Treatment means							
Surgery	3		1.45 (0.69–3.05)	0.33	45	0.16	Fixed-effects model
Other	3		3.61 (2.10–6.21)	<0.001	0	0.65	Fixed-effects model
Country							
Asia	2		2.45 (0.89–6.75)	0.08	0	0.32	Fixed-effects model
Other	4		2.34 (0.94–5.83)	0.07	86	<0.001	Random-effects model

HR = hazard ratio, OS = overall survival, TNM, tumor node metastasis classification; VEGF = vascular endothelial growth factor.

**Figure 3.**
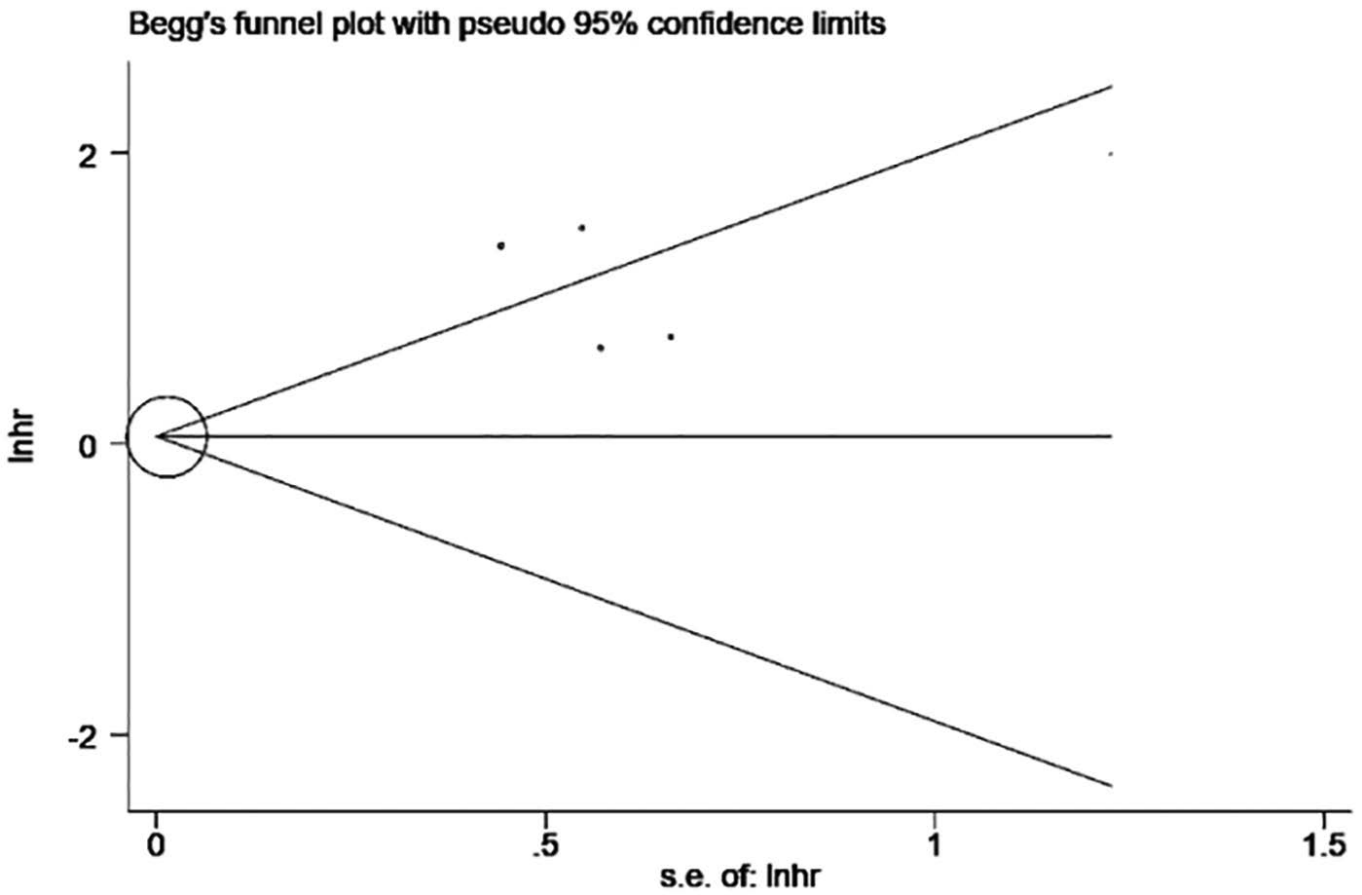
Funnel plot for assessing publication bias in OS. OS = overall survival, s.e.of:Inhr = standard deviation of lnhr.

**Figure 4.**
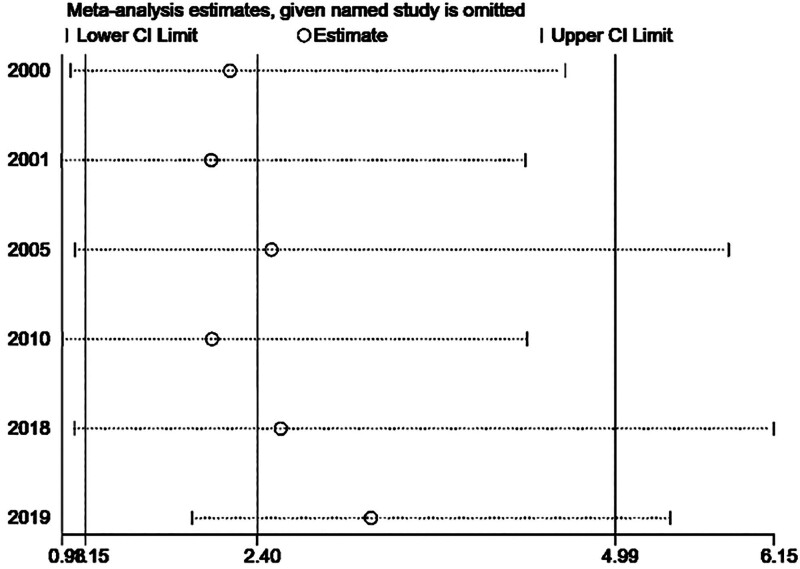
Sensitivity analysis of all the studies. CI = confidence interval.

Analysis by Begg test (*P* = 1.000) showed that there was no publication bias in our meta-analysis (Fig. 3). In addition, sensitivity analysis showed that all studies were stable (Fig. 4).

#### 3.3.2. DFS and DSS

**Figure 5.**
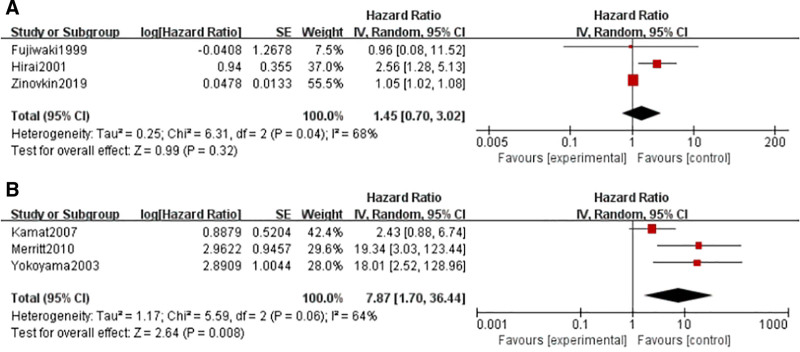
(A) Forest map of VEGF expression and disease-free survival of endometrial cancer. (B) Forest map of VEGF expression associated with DSS of endometrial cancer. CI = confidence interval, DSS, disease-specific survival, IV = random effects model, SE = standard deviation, VEGF = vascular endothelial growth factor.

Three studies reported DFS and DSS data, including 267 and 378 cases, respectively. As shown in Figure 5, high VEFG expression was significantly correlated with poor DSS (HR = 7.87, 95% CI = 1.70–36.44, I^2^ = 64%, *P* = .008), but no significant relationship was observed between high VEGF expression and DFS (HR = 1.45, 95% CI = 0.70–3.02, I^2^ = 68%, *P* = .32).

### 3.4. Relationship between VEGF expression and clinicopathological features of patients

We analyzed the correlation between VEGF expression and tumor stage, histological grade, lymph node status, and depth of myometrium infiltration (Table 4). Combined results showed that, compared with low VEGF expression, high VEGF expression was significantly associated with later tumor stage (*P* < .001), lower histological differentiation (*P* = .007), and earlier lymph node metastasis (*P* < .001). However, VEGF expression was not significantly associated with the depth of myometrial invasion (*P* = .14).

**Table 4:** Association between high VEGF expression and clinicopathological features

Clinicopathological characteristics	No. of studies	No. of patients	Pooled OR (95% CI)	*P* value	Heterogeneity		Model
					I^2^ (%)	*P* value	
TNM stage (IV-III vs I-II)	4	382	3.70 (2.22–6.19)	<0.001	17	0.30	Fixed-effects model
Histological grade (G3 vs G1-G2)	5	469	2.08 (1.22–3.55)	0.007	0	0.62	Fixed-effects model
Lymph node metastasis (yes vs no)	2	299	5.42 (2.35–12.47)	<0.001	0	0.33	Fixed-effects model
Myometrial invasion (>½ vs ≤½)	3	271	2.01 (0.79–5.11)	0.14	58	0.09	Random-effects model

OR = odds ratio, TNM = tumor node metastasis classification, VEGF = vascular endothelial growth factor.

## 4. Discussion

A large number of studies have proved that VEGF expression is important for the prognosis of patients with cancer. For example, in esophageal squamous-cell carcinoma, patients with decreased VEGF expression tend to have longer OS and progression-free survival.^[21]^ The 5-year survival rate of patients with cervical cancer with high levels of VEGF in tumor tissues is significantly lower than that of patients with low levels.^[22]^ However, the relationship between VEGF expression in endometrial cancer tissues and prognosis is still controversial in various studies; so we included a total of 11 studies, including a total of 1251 patients with endometrial cancer, to further evaluate the correlation between VEGF and survival outcomes in patients with endometrial cancer.

We finally identified 11 articles based on inclusion criteria, and by combining HR and 95% CI data, our findings showed that high VEGF expression in cancer tissues was associated with shorter OS and DSS in patients after surgery. This result is consistent with most of the findings in this meta-analysis. In addition, we found no significant association between VEGF expression and DFS in patients with endometrial cancer through our existing data analysis. Perhaps due to the limited data, it is not possible to demonstrate the influence of VEGF level on DFS at present. Therefore, a large number of future studies are needed to further evaluate the prognostic role of VEGF in DFS.

Cancer recurrence and metastasis often predict poor prognosis, and angiogenesis plays a crucial role in the process of cancer recurrence and metastasis. In this study, we also analyzed the relationship between VEGF expression and histopathological parameters of tumor invasiveness. We found that, compared with patients with low VEGF expression, patients with high VEGF expression had more advanced tumor stage, worse tissue differentiation, and were more likely to have lymph node metastasis, which was consistent with previous research results of Xu et al.^[23]^ These aggressive histopathological features may significantly affect the survival outcomes of patients with cancer. Surprisingly, we did not observe a significant relationship between VEGF expression and the depth of myometrial invasion. Due to the small number of included studies and significant heterogeneity between studies, the results may lack reliability and need to be interpreted with caution. Therefore, it is necessary to further explore the correlation between the high expression of VEGF and pathological features.

Our meta-analysis is the first to explore the prognostic value of high VEGF expression in endometrial cancer. It is well known that cancer proliferation, invasion, and metastasis are inseparable from angiogenesis, and VEGF is the most effective regulator in this process.^[24]^ As early as 2002,^[25]^ studies have reported that VEGF overexpression is correlated with angiogenesis and poor prognosis of patients with endometrial cancer. Later, Ma et al^[26]^ showed that VEGF expression was significantly elevated in patients with poorly differentiated, advanced and metastatic endometrial cancer, and Topolovec et al^[19]^ also reached a similar conclusion in their study. The significance of this meta-analysis is to provide a theoretical basis for VEGF as a useful indicator to evaluate the prognosis of patients with endometrial cancer. It has been shown that VEGF can predict disease recurrence in patients with stage II colon cancer and can be used to identify patients at high risk of recurrence who may benefit from adjuvant therapy.^[27]^ Liu et al^[28]^ performed risk scores by detecting protein kinase B, VEGF, and NIN1/RPN12 binding protein 1 homolog in resected non–small-cell lung cancer and found that the postoperative survival rate of resected non–small-cell lung cancer patients could be predicted based on the risk scores expressed by protein kinase B, VEGF, and NIN1/RPN12 binding protein 1 homolog. It can be seen that VEGF is meaningful for clinical prediction of the prognosis of certain cancers. Therefore, VEGF expression levels can be detected in cancer tissues after endometrial cancer surgery, alone or in combination with other markers, to help predict disease recurrence and adverse clinical outcomes.

There are some limitations to our study. First, there was significant heterogeneity in the combined results; so the results should be interpreted with caution. Second, part of HR and 95% CI data are derived from survival curve extraction, which is not as accurate as the original data directly provided, and some errors are unavoidable. Third, the number of included articles is small, which may affect the reliability of the results. Therefore, more and larger studies are needed to further explore in the future.

## 5. Conclusion

In conclusion, our meta-analysis suggests that, compared with low VEGF expression, high VEGF expression is significantly associated with poor survival outcomes (OS, DSS), later clinical stage, poorer differentiation, and lymph node metastasis in patients with endometrial cancer. VEGF may be an important marker for predicting the prognosis of endometrial cancer, which can help guide clinical treatment and follow-up. Due to some limitations, our findings need to be further validated by more high-quality studies with larger sample sizes.

Because the data used did not include personal data, ethical approval and patient consent are not required for this meta-analysis.

## Author contributions

**Writing—original draft:** Bao Qiang.

**Supervision:** SanYuan Zhang.

**Software:** YiFan Kang.

**Data curation:** JiaoLin Yang, ChunMei Zhang.

**Formal analysis:** HuanCheng Su.

**Methodology:** Zhe Wang.
